# What did CRISPR-Cas9 accomplish in its first 10 years?

**DOI:** 10.11613/BM.2023.030601

**Published:** 2023-08-05

**Authors:** Yehya Khlidj

**Affiliations:** University of Algiers 1, Faculty of Medicine, Algiers, Algeria

**Keywords:** CRISPR/Cas9, deoxyribonucleases, gene editing, genetic therapy

## Abstract

It’s been 10 years now from the debut of clustered regularly interspaced short palindromic repeats-associated protein 9 (CRISPR/Cas9) era in which gene engineering has never been so accessible, precise and efficient. This technology, like a refined surgical procedure, has offered the ability of removing different types of disease causing mutations and restoring key proteins activity with ease of outperforming the previous resembling methods: zinc finger nucleases (ZFNs) and transcription activator-like effector nucleases (TALENs). Additionally, CRISPR-Cas9 systems can systematically introduce genetic sequences to the specific sites in the human genome allowing to stimulate desired functions such as anti-tumoral and anti-infectious faculties. The present brief review provides an updated resume of CRISPR-Cas9’s top achievements from its first appearance to the current date focusing on the breakthrough research including *in vitro, in vivo* and human studies. This enables the evaluation of the previous phase ‘the proof-of-concept phase’ and marks the beginning of the next phase which will probably bring a spate of clinical trials.

## Introduction

Clustered regularly interspaced short palindromic repeats-associated protein 9 (CRISPR-Cas9) has become a turning point in the history of gene editing. Its first introduction was in 2012 by Jennifer Doudna and Emmanuelle Charpentier and their colleagues who demonstrated the therapeutic potential of CRISPR-Cas9 technology (*1*). This work was awarded in 2020 with Nobel Prize in Chemistry (*2*). Nevertheless, the idea of gene editing systems has emerged way before with the two approaches zinc finger nucleases (ZFNs) and transcription activator-like effector nucleases (TALENs) (*3*). Compared to these, CRISPR-Cas9 is simpler, less expensive, more efficient and has the advantage of multiplex genome engineering (*4*, *5*).

Basically, CRISPR-Cas9 is an anti-infectious mechanism that exhibit bacteria but not human cells to impede the insertion of invasive pathogens of their genomic sequences (such as plasmids, transposons or phage DNA) *via* the cleavage of the integrated foreign genome followed by proper repair of the host cell genetic material (*6*). When it is employed to produce desired genetic modifications or suppress/correct pathological mutations it allows a sophisticated manipulation of human genome. Consequently, CRISPR-Cas9 has been tested in a large number of preclinical studies and even entered the clinical trial phase recently as an investigated therapy for a few dramatically evolving diseases such as resistant cancers and incurable genetic disorders ultimately demonstrating first of their kind, promising results (*7*-*11*). This report briefly reviews the CRISPR-Cas9 journey in the past decade by shedding light on its highest accomplishments in both preclinical and clinical studies. Therefore, this can be a reference to evaluate the upcoming progresses.

## Methods

An extensive literature search was conducted *via* access to the search engines “PubMed”, “Scopus”, “Google Scholar” and “Google”. The following search keywords as well as their derivatives were entered in varying combinations: CRISPR-Cas9, gene editing, gene therapy, in-human trials, animal models, β-thalassemia, sickle cell disease, cancer, human immunodeficiency virus (HIV) infection, rare disease, transthyretin amyloidosis, hypercholesterolemia, Leber congenital amaurosis type 10, retinitis pigmentosa, Parkinson’s disease, Huntington’s disease, Alzheimer’s disease, amyotrophic lateral sclerosis, cystic fibrosis, alpha-1 antitrypsin deficiency, Duchenne muscular dystrophy, inborn errors of metabolism, and tyrosinemia type 1. Studies were included if they: (i) showed empirical results, and (ii) dated within the studied period (2012 to 2023). In total, 38 hallmark studies were identified from the literature, four of which included human patients. The studies’ protocols and results were thoroughly read to ensure accurate description.

## Principle and mechanism of action

Clustered regularly interspaced short palindromic repeats-associated protein 9 is originally an immune defence mechanism in prokaryotes against infective organisms like bacteriophages that can insert their DNA sequences “protospacers” into the host cell genome. The fragments of the invading DNA termed “spacers” are integrated into CRISPR array which is formed of identical short repeat sequences interspaced by pathogens-derived spacers. The CRISPR array is then transcribed to pre-CRISPR RNA (pre-crRNA) which matures to CRISPR RNA (crRNA), and the latter is base-paired with a hairpin RNA known as trans-activating crRNA (tracrRNA), which serves as trigger of pre-crRNA maturation by RNase III and activator of crRNA-guided DNA cleavage by CRISPR associated (Cas) 9 nuclease. The crRNA-tracrRNA interaction will allow the formation of a dual-RNA guide, sometime called gRNA (guide RNA) that directs the activity of Cas9 protein. As a result, the complex crRNA, tracrRNA and Cas9 nuclease becomes able to recognize the foreign DNA through crRNA and cleave (silence) it through Cas9 (*1*, *6*, *12*, *13*). Cas9 has two nuclease domains, the HNH which cleaves the complementary strand, and the RuvC-like domain which cleaves the non-complementary strand (*1*). There are six types of CRISPR systems divided according to the size, the site of cleavage and the protospacer adjacent motif (PAM) region of Cas nucleases (Cas3, Cas9, Cas12a, and others). CRISPR-Cas9 corresponds to type II system in which the PAM region of Cas9 is a 5’-NGG-3’ sequence that is located at the 3’ end of the target sequence (*13*). Due to its wide range of therapeutic potential CRISPR-Ca9 has been transformed into a gene repair tool adapted from bacterial systems such as that of *Streptococcus pyogenes* to allow targeting specific genome sequences (*e.g*., the locus in human genome that holds a disease causing mutation) and editing them (*14*, *15*). After recognition of the target DNA region by a complementary crRNA, the nuclease Cas9 induces a nick or double-strand breaks (DSBs) at this site subsequently activating cellular genome repair pathways. There are two main mechanisms of DNA DSBs repair, non-homologous end joining (NHEJ) and homology-directed repair (HDR) (*16*). While HDR fills the nuclease created breaks with homologous donor DNA, NHEJ is an error-prone process that re-joins the ends of the broken DNA together without replacing the lost sequences which may result in a heterogeneous repaired DNA often containing insertions and deletions (*14*, *17*).

Therapeutic CRISPR-Cas9 differs from the original bacterial CRISPR-Cas9 by fusing the tracrRNA-crRNA duplex in form of one RNA chimera “sgRNA” which produces a single RNA-guided Cas9 system that can be programmed for site-specific DNA cleavage and subsequent genome engineering (*1*). In preclinical studies, two main strategies are used. First, the *in vivo* approach that targets zygote or adult somatic cells and acts by directly infusing the CRISPR-Cas9 components including Cas9 messenger RNA or nuclease, sgRNA and HDR or NHEJ template to the patient through a delivery system (*14*). The latter can be of a viral nature (the most commonly used carrier) or a non-viral carrier such as the recently emerged nanomaterials (cationic lipid nanoparticles (LNPs), DNA nanoparticles, lipid complexes, gold-based nanoparticles, and zeolite imidazole framework) (*18*). The other strategy is *ex vivo* process in which CRISPR-Cas9 mediated gene editing occurs in patient-derived pluripotent stem cells which leads to the generation of clones of reprogrammed or genetically corrected progenitors that are then transplanted to the patient where they differentiate to produce adult somatic cells exhibiting the wanted genetic features (*14*).

Genetic editing can aim either to knock-in or knock-out the target genes. Knock-in genome repair consists of inserting small nucleotide sequences, co-injected with the CRISPR-Cas9 machinery, which are incorporated into the DNA breaks typically through HDR to substitute the Cas9 cleaved sequences. This approach can be used for therapeutic intentions mainly the correction of pathogenic mutations either by repairing a gene *via* short oligonucleotides addition or by replacing it with a functional one (*19*). An example of this is the experiment performed by Chen *et al.* in which a *Staphylococcus aureus* Cas 9-guide RNA (SaCas9-gRNA), targeting the albumin (*Alb*) locus in liver cells DNA, and a codon-optimized human B domain deleted-FVIII (BDD-FVIII) were administered separately in haemophilia A mice models using adeno-associated virus (AAV) vectors (*20*). Consequently, the BDD-FVIII was inserted (knocked in) in hepatic cells *Alb* locus and was expressed as a functional FVIII protein.

By contrast, the knock-out approach allows the suppression (loss of function) of a coding gene. This can be accomplished through the indels (insertions or deletions) generated by the NHEJ induced errors when repairing the DSBs, creating frame-shift mutations with subsequent premature translation-termination codons leading to incomplete mRNA that is degraded by nonsense-mediated mRNA decay (*21*). Therefore, this mechanism is limited by the risk of inducing off-target mutations (*21*). Nevertheless, in many cases it can provide very promising results. One potential therapeutic use of such method is the knock-out of inhibitory checkpoint gene *PD-1* (programmed death-1), an inhibitor of T-cell activity that is stimulated by dendritic cells and cancer cells through *PD-L1* (PD-1 ligand), which upregulates the T-cell cytotoxicity and interferon-gamma (IFN-γ) production giving the possibility of a novel strategy for anti-tumoral immunotherapy (*22*). Interestingly, instead of directly administering the sgRNA and Cas9 protein, in the above study the authors used coding plasmids that were co-transfected to the primary T cells *via* electroporation.

## Summary on biggest therapeutic achievements

During the past decade several *in vitro*, *in vivo* and in human studies have been conducted to test the effectiveness of CRISPR-Cas9 with the majority showing satisfying and promising results. The below section will provide a non-exhaustive description of the most remarkable published studies from human to *in vivo/in vitro* trials concerning different groups of human illnesses. A summary of the upcoming section is provided in Table 1.

**Table 1:** Summary of CRISPR-Cas9 top achievements and proof-of-concept evidence in its first decade of testing

**Disease**	**Design**	**Target gene**	**Outcomes**	**References**
β-thalassemia and sickle cell disease	In human phase I trial	*BCL11A*	Increased concentrations of fetal haemoglobin (HbF)	(*7*, *8*)
Sickle cell disease	*Ex vivo*	β-globin gene	Synthesis of wild-type haemoglobin	(*23*)
Resistant CD19+ B cell acute lymphoblastic leukemia	In human phase I trial	Anti-CD19 chimeric antigen receptor	Generation of universal T cell populations that target tumour cells, leading to negative flow cytometry	(*9*)
Multiple refractory solid cancers including colorectal cancer, hormone-receptor-positive breast cancer, ovarian cancer, melanoma, and lung cancer	In human phase I trial	Mutational neoantigens-related genes	Achievement of disease stability in about third of the subjects	(*10*)
Orthotopic glioblastoma	*In vivo*	*PLK1*	Reduction in primary and metastatic tumour growth and significant increase in mice survival	(*24*)
Transthyretin amyloidosis	In human phase I trial	*TTR*	Significant drop in TTR protein	(*11*)
Cystic fibrosis	*In vitro*	*CFTR*	Increase in functional CFTR by more than 70%	(*25*, *26*)
Alpha-1 antitrypsin deficiency	*In vivo*	*SERPINA1*	Decrease in the expressed mutation (Pi*ZZ) and its related phenotypic features	(*27*-*29*)
Duchenne muscular dystrophy	*In vivo*	*DMD* gene	Restoration of functional dystrophin with subsequent improvement in muscular contractility	(*30*-*33*)
HIV-1 infection	*In vivo*	endogenous Igh locus in B cells	Induction of sustainable humoral response	(*35*)
	*In vivo*	*Gag*	Elimination of integrated proviral DNA and clearance of viremia	(*36*)
	*In vitro*	*tat* and *rev*	Suppression of viral infection	(*37*)
	*In vitro*	*CCR5*	Induction of indels in the CCR5 protein	(*38*)
Tyrosinemia type 1	*In utero* of mice models	*HPD*	Survival of Fah–/– mice and amelioration in liver function	(*40*)
Autosomal dominant hypercholesterolemia	*In vivo*	*PCSK9*	Decrease in LDL cholesterol concentrations	(*42*-*44*)
	*In vivo*	*LDLR*	Restoration of wild-type LDLR, and decrease in atherogenic dyslipidemia as well as pathological features of atherosclerosis	(*45*)
	*In vitro*	*LDLR*	Permanent repair of homozygous deletion in LDLR gene	(*46*)
Leber congenital amaurosis type 10	*In vitro* and *in vivo*	*CEP290*	Correction of the disease-causing mutation	(*48*, *49*)
	*In vitro*	*MAK*	Restoration of the retinal transcript and protein in patient cells	(*50*)
Retinitis pigmentosa	*In vivo*	*RPGR*	Expression of full length RPGR ORF15 protein and disappearance of hallmark features of RPGR mutation	(*51*)
Amyotrophic lateral sclerosis	*In vitro*	*SOD1* and *FUS*	Correction of SOD1 mutation in patient iPSCs	(*52*)
	*In vivo*	*SOD1*	Increase in motoneurons, delay in disease onset, and prolongation in lifespan	(*53*)
	*In vitro* and *in vivo*	*C9ORF72*	Reversal of major disease mechanisms	(*57*)
Huntington’s disease	*In vivo*	*HTT*	Decrease in neurotoxic inclusions, increase in survival and partial recovery in motor dysfunction	(*54*)
Parkinson’s disease	*In vitro*	*LRRK2*	Resuscitation of parkinsonism phenotypes in iPSC-derived dopaminergic neurons	(*55*)
	*In vitro* and *in vivo*	*SNCA*	Reduction of α-synuclein overexpression, reactive microgliosis, dopaminergic neurodegeneration, and parkinsonian motor symptoms	(*58*)
Alzheimer’s disease	*In vivo*	*App*	Down-expression of amyloid precursor protein	(*59*)
	*In vitro*	*PSEN1*	Partial restoration of amyloid-β 42/40 in human fibroblasts carrying PSEN1 mutation	(*56*)
CRISPR-Cas9 - clustered regularly interspaced short palindromic repeats-associated protein 9.				

### Inherited haemoglobinopathies

Finding a curative treatment for inherited haemoglobinopathies, mainly thalassemia and sickle cell disease, has always been an elusive goal. In 2021, Frangoul *et al.* reported the first cases of transfusion-dependent β-thalassemia patient and sickle cell disease patient effectively treated by CRISPR-Cas9-based gene editing (*7*). In this study, patients received autologous CRISPR-Cas9-edited CD34+ haematopoietic stem and progenitor cells that were genetically modified by targeting *BCL11A* expression (a repressor of γ-globin expression) to produce higher concentrations of foetal haemoglobin (HbF) in adult erythrocytes. With the same principle post-transcriptional genetic silencing of *BCL11A* was applied in six patients with sickle cell disease leading to broad distribution of HbF with significant reduction or absence of clinical manifestations (*8*). Moreover, using CRISPR-Cas9 technology, Hoban *et al.* induced precise correction of sickle cell disease mutation in human haematopoietic stem cells which resulted in the synthesis of wild-type haemoglobin (*23*).

### Refractory cancers

CRISPR-Cas9 has rapidly attracted attention as a potential game-changer weapon in the field of oncology and immunotherapy due to its sophisticated potentials of cells reprogramming. One trending idea is to modify genome of cytotoxic T cells in the sake of enhancing their ability of tumour shrinkage. In 2022, a phase I open-label non-randomized clinical trial involved six children with relapsed and treatment resistant CD19+ B cell acute lymphoblastic leukaemia that were treated with next-generation CRISPR-Cas9 engineered immunotherapy (*9*). The latter was based on the “off-the-shelf” generation of anti-CD19 chimeric antigen receptor (CAR19) universal T cell populations. After lymphodepleting chemotherapy, patients received single infusion of the CAR19 T cells. Remarkably, four of them showed cell expansion and negative flow cytometry, and then underwent allogeneic stem cell transplantation. Major adverse events included the development of grade II cytokine release syndrome in two patients, transient grade IV neurotoxicity in one patient and manageable skin graft-*versus*-host disease in other patient, nevertheless, the overall safety profile was acceptable. In another recent in-human phase I clinical trial, Foy *et al.* demonstrated the potential of CRISPR-Cas9 of producing clones of genetically-engineered T cells that are able to target tumour cells mutational neoantigens through the generation of neosynthetized tumour-specific T cell receptors (neoTCR) (*10*). They then infused the produced cells in sixteen subjects who had multiple refractory solid cancers (colorectal cancer, hormone-receptor-positive breast cancer, ovarian cancer, melanoma, and lung cancer) after they underwent chemotherapy induced lymphopenia. Notably, about third of the patients (31%) had stable disease while the remaining had their disease progressed. One patient developed grade I cytokine release syndrome and another had grade III encephalitis. Furthermore, a novel LNPs CRISPR-Cas9 formulation (sgPLK1-cLNPs) had permitted a safe and effective (~70%) gene editing *in vivo* of aggressive orthotropic glioblastoma cells by targeting their *PLK1* (polo-like kinase 1) gene (*24*). This induced apoptosis of cancer cells, reduced tumour growth by 50% and increased survival in treated mice. Additionally, when coupled with the epidermal growth factor receptor (EGFR) - targeting antibodies, sgPLK1-cLNPs was systemically and selectively disseminated to reach metastatic ovarian cancer cells allowing ~80% of gene editing, significant tumour growth inhibition, and 80% increase in survival.

### Rare incurable disease

Transthyretin amyloidosis is a hereditary incurable disease caused by misfolded transthyretin (TTR) proteins build-up mainly affecting the nervous and cardiac tissues. In a remarkable phase I clinical trial, CRISPR-Cas9-sytem based agent (NTLA-2001) knocked-out the *TTR* mutant when injected into a small group of patients provoking reduction in TTR protein concentration by 52% with a dose of 0.1 mg/kg, and 87% with a dose of 0.3 mg/kg, at day 28 (*11*). CRISPR-Cas9 also repaired the cystic fibrosis (CF) causative mutations (*i.e*., *CFTR* deletion) in human organoids cells (*25*) (*25*). CRSIPR-Cas9 mediated insertion of full *CFTR* coding DNAs in human airway basal stem cells obtained from donors with CF had led to an increase in functional CFTR more than > 70% compared to that observed in corrected non-CF donor’s cells (*26*). Regarding alpha-1 antitrypsin (AAT) deficiency, a hereditary disease caused by *SERPINA1* (serine protease inhibitor A1) mutation that can potentially evolve to liver and lung failure, CRISPR-Cas9 approach was used for *in vivo* correction of severe forms linked mutation (*i.e*., Pi*ZZ) in transgenic mouse models (*27*). In this experiment, the treated mice exhibited substantial (> 98%) reduction in *AAT-Z* mutant expression and a modest (5%) restoration of *AAT* wild-type, *AAT-M*. The capability of CRISPR-Cas9 to rectify *AAT-Z* mutation and partially induce *AAT* wild-type expression was further demonstrated in *in vivo* studies (*28*, *29*). Besides eliminating *SERPINA1* mutation and its relevant products, a single dose of CRISPR-Cas9 therapy significantly decreased pathological features of liver inflammation and fibrosis in humanized mouse models (*29*). Another involved disease in CRISPR-Cas9 research was Duchenne muscular dystrophy (DMD), a severe X-linked myopathy caused by mutations in the human *DMD* gene which results in a defective dystrophin protein. Recently, repair attempts of these mutations with CRISPR-Cas9 mediated gene editing have become a popular strategy by numerous studies (*30*-*33*). For example, one common mutation is a deletion of exon 44 that leads to subsequent generation of a premature termination codon in exon 45. To correct this mutation, mice models of DMD were treated with Cas9 nuclease and sgRNA delivered through a dual AAV (*30*). Post-intervention examination revealed restoration in expression of dystrophin in a dose dependent manner along with improvement in muscular contractility.

### HIV-1 infection

Since the attempts of CRISPR-Cas9 application are extended to multiple knotty diseases, HIV infection is not an exception. Different strategies are being tested such as eradication of the integrated latent provirus, reactivation of dormant virus to enhance infected cells natural death “shock and kill”, and prevention of viral infection through induction of neutralizing antibodies or *via* avoidance of viral entry by modification of CD4 co-receptor, CCR5 (*34*, *35*). The combination of sequential long-acting slow-effective release antiviral therapy (LASER ART) with CRISPR-Cas9 induced the elimination of HIV-1 in infected humanized mice (*36*). Furthermore, targeting HIV-1 regulatory genes *tat* and *rev* by CRISPR-Cas9 in T-cell lines exhibiting persistent and latent HIV-1 infection elicited inhibition of viral infection, manifesting as suppression of p24 levels (*37*). In different experiment, CRISPR-Cas9 induced indels in the *CCR5* locus of human cells at significant frequencies ranging from 5% to 33% (*38*). Development of broadly neutralizing antibodies against HIV-1 was also possible with CRISPR-Cas9 edited B cells and this offered sustainable specific humoral response in wild-type mice that received the protective antibodies (*35*).

### Inborn errors of metabolism

Inborn errors of metabolism (IEM) are a significant source of child morbidity and mortality worldwide, with a fatality rate of ≥ 33%, causing at least 23,529 deaths each year globally (0.4% of all global child deaths) (*39*). This group of disease is usually due to monogenic mutations that lead to enzymes/metabolism-involved proteins deficiencies which in turn provoke toxic accumulation of non-catabolized substrates in different tissues. Tyrosinemia type 1 is a monogenic IEM secondary to *Fah1* gene mutations which can be partially stabilized by upregulation of enzyme 4-hydroxyphenylpyruvate dioxygenase (HPD) activity. *In utero* targeting of HPD rescued the lethal phenotype in *Fah*^–/–^ mice and improved liver function (*40*). Autosomal dominant hypercholesterolemia (ADH), another form of IEM that can be caused by mutations in the *LDLR* (low-density lipoprotein receptor) gene, the *APOB* (apolipoprotein B) gene or the *PCSK9* (proprotein convertase subtilisin/kexin type 9) gene. The latter was recently found to be associated with ADH as it was found to encode for neural apoptosis regulated convertase (NARC-1) which is an enzyme highly expressed by liver cells and is strongly implicated in the cholesterol homeostasis (*41*). *In vivo* knockdown of hepatic *PCSK9* by infused CRISPR base editors caused a decrease in PCSK9 serum concentrations by ~90% and dropped low-density lipoprotein (LDL) cholesterol concentrations by ~60% in primate models (*42*). In fact, several preclinical studies have been conducted to test the effectiveness of CRISPR-Cas9 and showed its long-term potential to maintain a stable gene editing and concomitantly reduce circulating PCSK9 and LDL cholesterol (*43*, *44*). In addition, *LDLR* gene can also be a prominent target of CRISPR technologies. Zhao *et al*. induced *in vivo* correction of *LDLR* mutation in mice hepatocytes through the administration of CRISPR/Cas9 system delivered by AAV (*45*). Hence, this was associated with partial restoration in LDLR protein, significant reduction in blood concentrations of total cholesterol, total triglycerides, and LDL cholesterol, as well as decrease in the size of atherosclerotic plaques and infiltration of macrophage in the aorta. Moreover, Omer *et al.* successfully repaired a 3-base pair homozygous deletion in the exon 4 of *LDLR* gene in patient-derived pluripotent stem cells which allowed subsequent generation of hepatocyte-like cells that exhibited normal LDLR-mediated endocytosis (*46*).

### Inherited retinal degenerations

Inherited retinal degenerations (IRD) are a heterogeneous group of inherited disorders induced by genetic mutations affecting retinal function and manifest with progressive visual impairment possibly leading to blindness (*47*). Studies have shown the promising effects of CRISPR-Cas9 in removing the genetic error involved in Leber congenital amaurosis type 10, an IRD caused by *CEP29* gene mutations which leads to severe retinal dystrophy and loss of vision (*48*, *49*). Thus, Ruan *et al.* were able to restore the expression of *CEP29* gene wild-type through CRISPR-Cas9 induced deletion of intronic splice mutation, the most frequent mutation of *CEP29* (*48*). Moreover, in the context of IRD, the correction of disease-causing mutations in patient-derived induced pluripotent stem cells (iPSCs) would enable their transplantation in form of autologous cell replacement therapy (*50*). Retinitis pigmentosa, another IRD often caused by frameshift mutations in the *OFR15* exon of *RPGR* (retinitis pigmentosa GTPase regulator) gene. Mice carrying this mutation when genetically edited with CRISPR-Cas9 exhibited disappearance of *RPGR* mutation relevant features including mislocalization of opsins in photoreceptor cells (*51*).

### Neurodegenerative disorders

Lately, CRISPR-Cas9 utility has been investigated in animal models of neurodegenerative diseases that are induced by -or associated with- genetic abnormalities such amyotrophic lateral sclerosis (ALS), Huntington’s disease (HD), Parkinson’s disease (PD) and Alzheimer’s disease (AD) in the purpose of paving the way for in-human trials (*52*-*56*). Familial ALS is known to be caused by *SOD1* (superoxide dismutase 1) and *FUS* (fused in sarcoma) mutations. Studies showed the ability of CRISPR-Cas9 in restoring the wild-type of these genes either *in vitro* or *in vivo* (*52*, *53*). In one study, correction of *SOD1* mutant gene mediated by CRISPR-Cas9 therapy increased motoneurons number, delayed disease onset and prolonged survival in ALS mice (*53*). A recent work demonstrated successful *in vivo* excision of hexanucleotide repeat expansion in *C9ORF72* gene, the most frequent genetic cause of ALS and frontotemporal dementia, by CRISPR/Cas9 machinery (*57*). Further, CRISPR-Cas9 system viro-delivered to the striatum of HD mice enabled the disruption of CAG trinucleotide repeat in exon 1 of the *HTT* (huntingtin) gene (the mutation responsible of HD) (*54*). Notably, this was associated with about 50% drop in neurotoxic inclusions, about 15% increase in lifespan and a significant improvement in certain motor functions. As for autosomal dominant PD, deletions of synuclein alpha (*SNCA*) (encodes for α-synuclein protein) and leucine-rich repeat kinase 2 (*LRRK2*) mutant (responsible for late onset-PD) were the target strategies of recent studies (*55*, *58*). CRISPR-Cas9 engineered isogenic iPSCs to produce surviving dopaminergic neurons with reduced LRRK2 kinase activation and phospho-α-synuclein expression (*55*). Similarly, *in vitro* and *in vivo* deletion of *A53T* mutation in *SNCA* gene diminished the expression of α-synuclein, rescued dopaminergic neurons degeneration and ultimately relieved symptoms of parkinsonism (*58*). Additionally, Wang *et al.* designed a novel blood-brain barrier bypass strategy with glutathione (GSH)-responsive silica nanocapsules (SNCs) transport platform to optimize the delivery of CRISPR genome editors to different central nervous system genes such as amyloid precursor protein (*App*), the one involved in AD (*59*). Hence, following *in vivo* administration of SNCs transported CRISPR-Cas9, the expression of *App* gene was reduced by 19.1% in wild-type mice. Early-onset autosomal dominant AD is related to mutation in *PSEN1* (presenilin 1) gene, a promoter of amyloid-β (Aβ) peptide production and aggregation. Selective suppression of *PSEN1* mutation with CRISPR-Cas9 provoked reduction in Aβ proteins formation as well as presenilin 1 expression in mutation carrying human fibroblasts (*56*).

## What should be done in the next 10 years?

The main current critics for CRISPR-Cas9 when used for therapeutic purposes include suboptimal precision of the genome edition, risk of either undesired effects such as inducing insertions, large deletions, and chromosomal rearrangements, or off-target modifications (the most prominent disadvantage), restriction of activity to sites that contain a specific PAM, risk of immune degradation of Cas9 proteins, irreversibility of genomic mutagenesis, and lack of in human testing for adequate assessment of both efficacy and safety profile (*60*, *61*).

The off-target effect is the induction of mutations at sites other than the desired on-target site. This is a major problem of CRISPR-Cas9 which occurs frequently in more than 50% of the cases (*62*). The off-target sites are often sgRNA-dependent since the sgRNA comprises the seed sequence which is the one that determines the specificity of Cas9, however, sgRNA-independent off-target effects also exist (*62*, *63*). Such unintended cleavage might lead to the dysfunction or dysregulation of non-targeted genes which, when occur, can impede the progression to human trials phase. Höijer *et al.* demonstrated that CRISPR-Cas9 induced off-target effects can lead to large structural variants in the affected genes, and the resulted mutations segregate across generations (*64*). Moreover, the identification of off-target cleavage by CRISPR-Cas9 nucleases is difficult due to reduced sensitivity of the previously developed detection tools (*62*). The requirement of PAM near the target site is also another important issue of CRISPR-Cas9 that needs to be resolved. Thus, longer PAM sequences such as that of *Staphylococcus aureus* Cas9 (“5'NNGRRT3' or 5'NNGRR(N)3'”, with R can be any purine) make the window of therapeutic targeting sites more narrowed, thereby, reducing the amount of genetic editing and increasing the risk of missing the targeted genome (*65*). Furthermore, CRISPR-Cas9 induced DNA modifications often cause cell apoptosis by secondary activation of p53 in response to DSBs (*66*). In one experiment, Ihry *et al.* showed that high efficiency of indels generation by Cas9 was associated with lethal toxic effects in most human pluripotent stem cells (*66*). When used as cancer therapy, CRISPR-Cas9 may have higher risk of inducing undesired mutations in case of pre-existing mutations in cancer-related genes like *TP53* and *KRAS* (*67*). An additional problem is that the introduction of CRISPR-Cas9 system can trigger an immune response against Cas9. This is due to the bacterial nature of these proteins which are derived from the common lifelong pathogens *Streptococcus pyogenes* and *Staphylococcus aureus*. As a consequence, there is a substantial risk of pre-existing immunity against bacterial Cas9 proteins that can lead to their rapid degradation upon the injection (*61*). The unpredictable and broad-reaching effects of CRISPR-Cas9 may also create an ethical debate and social controversy, especially with the potential implications of this technique in human germline modification which raises concerns about threats to human integrity and dignity (*61*).

If CRISPR-Cas9 technology could overcome these limitations, it would undoubtedly mark the birth of new era of medicine where objectives for treatment of genetically determined diseases can be so ambitious. This probability should motivate researchers in the next decade to continue investigating the efficiency of such technology as well as its security, and aim to provide more precise and error-free genetic engineering results. Nonhomologous end joining off-target effects can be prevented by careful determination of target locus, refine selection of the sgRNA and effective and precise delivery method (*21*). Moreover, the suppression of unwanted editing can be achieved by co-administration of dead-RNAs and catalytically inactivating truncated gRNAs that allows prevention of adverse HDR mediated cleavage with preservation of on-target editing (*68*). The prediction of off-target activity is now possible through *in silico* tools and experimental methods (*63*). Besides prediction of unwanted gene editing, bioinformatics tools enable the design of more accurate gRNA significantly enhancing the specificity of CRISPR-Cas9 systems (*61*). Instead of normal Cas9 protein, the use of Cas9 nickases which have a less damaging mechanism to target DNA, can minimize the number of off-targeting (*69*). Another strategy to reduce unwanted gene editing is to inactivate Cas9 protein once it targets its site through the administration of anti-CRISPR proteins (Acr) (*61*). Dependency to PAM restricted genome recognition can be avoided by PAM-free nucleases through ortholog mining and protein engineering (*70*). Notable, newly engineered Cas9 can broaden the human targeting sites by 2- to 4-fold (*65*). The immune response against endogenous Cas9 protein can be prevented by targeting immune-privileged organs (eyes, brain, placenta, foetus, uterus and testicles) where there is a low risk of immunological rejection, or by gene editing at early life prior to the development of anti-Cas protein response (*61*). Potentials of genotoxicity should be rigorously monitored throughout the different stages of clinical trials and benefit risk ratio of gene editing therapy should always be by far in favour of clinical response. The preservation of morality concepts should be a priority for scientists during the use of gene editing technology, and experiments on humans should only be executed after strictly obtaining the proof of concept from animal studies. Regarding the economic cost, unlike ZFNs and TALENs, CRISPR-Cas9 systems are much cheaper, which is another pertinent reason to pursue the scientific investment in such potentially highly cost-effective therapy (*5*).

## Conclusions

The first decade of CRISPR-Cas9 research has provided the proof-of-concept for further advanced testing of this strategy. Thus, it showed that CRISPR-Cas9 can be a disease modifying approach for an increasing number of diseases *a priori* those with a genetic pathogenic base. These include cancers, inherited proteins deficiencies and enzymopathies, IEM, neurodegenerative disorders, and perhaps any disease caused by monogenic mutations. Moreover, it has the potency to enhance the immune system’ abilities to fight pathogens and might be a unique preventive tool for infectious diseases such as HIV in at risk individuals. Yet, significant barriers still exist mainly the off-target effects and lack of in-human trials. The next step therefore should focus on optimizing the precision of CRISPR-Cas9 technology as well as expanding the evidence of effectiveness and safety by carefully and progressively involving human patients.

## Data Availability

No data was generated during this study.
